# Impact of the Moderating Effect of National Culture on Adoption Intention in Wearable Health Care Devices: Meta-analysis

**DOI:** 10.2196/30960

**Published:** 2022-06-03

**Authors:** Zhenming Zhang, Enjun Xia, Jieping Huang

**Affiliations:** 1 School of Management and Economics Beijing Institute of Technology Beijing China

**Keywords:** wearable health care devices, national culture, moderating effect, meta-analysis

## Abstract

**Background:**

Wearable health care devices have not yet been commercialized on a large scale. Additionally, people in different countries have different utilization rates. Therefore, more in-depth studies on the moderating effect of national culture on adoption intention in wearable health care devices are necessary.

**Objective:**

This study aims to explore the summary results of the relationships between perceived usefulness and perceived ease of use with adoption intention in wearable health care devices and the impact of the moderating effect of national culture on these two relationships.

**Methods:**

We searched for studies published before September 2021 in the Web of Science, EBSCO, Engineering Village, China National Knowledge Infrastructure, IEEE Xplore, and Wiley Online Library databases. CMA (version 2.0, Biostat Inc) software was used to perform the meta-analysis. We conducted publication bias and heterogeneity tests on the data. The random-effects model was used to estimate the main effect size, and a sensitivity analysis was conducted. A meta-regression analysis was used to test the moderating effect of national culture.

**Results:**

This meta-analysis included 20 publications with a total of 6128 participants. Perceived usefulness (*r*=0.612, *P*<.001) and perceived ease of use (*r*=0.462, *P*<.001) positively affect adoption intention. The relationship between perceived usefulness and adoption intention is positively moderated by individualism/collectivism (β=.003, *P*<.001), masculinity/femininity (β=.008, *P*<.001) and indulgence/restraint (β=.005, *P*<.001), and negatively moderated by uncertainty avoidance (β=-.005, *P*<.001). The relationship between perceived ease of use and adoption intention is positively moderated by individualism/collectivism (β=.003, *P*<.001), masculinity/femininity (β=.006, *P*<.001) and indulgence/restraint (β=.009, *P*<.001), and negatively moderated by uncertainty avoidance (β=-.004, *P*<.001).

**Conclusions:**

This meta-analysis provided comprehensive evidence on the positive relationship between perceived usefulness and perceived ease of use with adoption intention and the moderating effect of national culture on these two relationships. Regarding the moderating effect, perceived usefulness and perceived ease of use have a greater impact on adoption intention for people in individualistic, masculine, low uncertainty avoidance, and indulgence cultures, respectively.

## Introduction

### Background

A wearable health care device can be defined as “an autonomous, noninvasive device that can perform specific medical functions such as long-term monitoring or improving health” [1]. The device can detect important vital indicators, such as heart rate, and enables rapid and remote autonomous detection and self-management of arrhythmia. These data can also be transmitted to medical institutions to achieve the purpose of remote health monitoring, thereby effectively reducing the number of patient visits and medical costs [2].

Since the outbreak of COVID-19, people have paid increasing attention to health, and the adoption of wearable health care devices is gradually increasing [3,4], but these devices have not yet been commercialized on a large scale. Therefore, it is necessary to conduct in-depth research on the factors that influence the adoption of wearable health care devices to promote the commercialization of the devices.

Many studies have examined adoption intention toward wearable health care devices [5-7]. These studies have mostly adopted the technology acceptance model (TAM) [8,9] and the unified theory of acceptance and use of technology (UTAUT) [5,6] as the main frameworks. In addition to the variables included in TAM and UTAUT, other variables such as trust [9-11], perceived privacy risk (from the privacy calculus model) [1,12], and consumer innovation (from the theory of innovation diffusion) [9,13] have been considered in the literature. Of the two models, TAM is the most concise and influential model [14] and provides a basis for tracing the influence of external factors on adoption intention. This model discusses the relationship between perceived usefulness, perceived ease of use, and adoption intention [15]. It is easy to understand, with information technology features, a strong theoretical foundation, and sufficient empirical support [16-20].

Studies that used this as the main model to analyze wearable health care device adoption intention, however, did not form a unified understanding, and there were conflicting conclusions on the relationship between perceived ease of use and adoption intention. Many studies have empirically confirmed this relationship [8,21,22]; however, some results have shown that this effect is not obvious [13,23]. Some studies have specifically explored the differences in conclusions caused by moderator variables in population characteristics and focused on the influence of different ages [8,24], genders [9], and experiences [25] on adoption intention in wearable health care devices to promote further commercialization of the devices in people with lower acceptance rates. Moreover, scholars have discovered that national culture also affects wearable health care device adoption intention [6,26], and large differences exist in the utilization rate of wearable health care devices in different countries [27]. Although the study by Meier et al [27] pointed out that under different cultural dimensions there are differences in wearable health care device use, it did not concentrate on how each cultural dimension affects adoption intention.

In view of the inconsistent conclusions in the existing studies and the insufficient exploration of the moderating effect of national culture, this study explores summary results of the relationships between perceived usefulness and perceived ease of use in wearable health care device adoption intention and the impact of the moderating effect of national culture on adoption intention by using the meta-analysis method. The results of this study could have implications for global wearable health care device providers in developing and marketing their devices successfully across borders, for effective enhancement of people’s health conditions, and for national health agencies to decrease medical expenses.

### Theoretical Framework and Hypotheses

#### Research Framework

**Figure 1:**
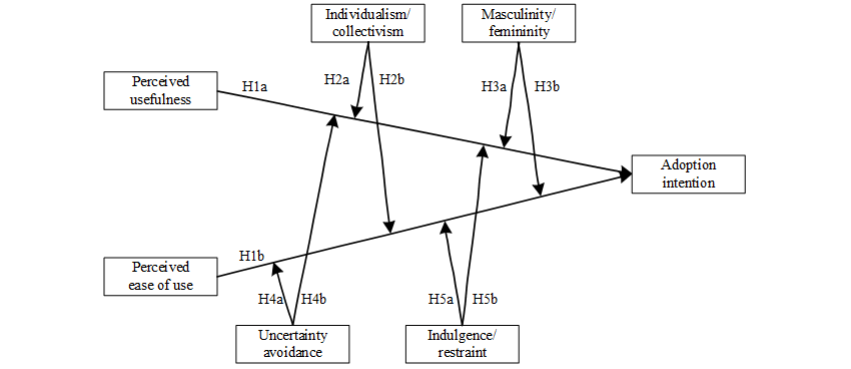
Research framework. H: hypothesis.

The research framework used in this study is presented in Figure 1. We chose TAM as the main model and Hofstede’s cultural value dimensions to represent national culture.

As mentioned above, TAM is the most concise and influential of the models with a strong theoretical foundation and sufficient empirical support [14,16-20]. The dimensions used to analyze cultural value mainly come from Rokeach [28], Hanson [29], and Hofstede [30]. The dimensions developed by Hofstede are the most recognized and commonly used framework for studying cross-cultural issues on technology adoption [31-34]. The formation process of the value of the cultural dimension has “a rigorous research design, a systematic data collection, and a coherent theory to explain national variations” [35], achieving the aggregation of the properties of individuals as observed within a country. Therefore, every cultural dimension can be treated as a country-level variable [36]. Hofstede’s cultural value contains 6 dimensions: power distance, individualism/collectivism, masculinity/femininity, uncertainty avoidance, long-term/short-term orientation, and indulgence/restraint [30]. This study focuses on the moderating effects of 4 of these: individualism/collectivism, masculinity/femininity, uncertainty avoidance, and indulgence/restraint.

First, power distance refers to the degree to which people accept an unequal distribution of power [37]. When commodities can represent the differences in the identity and power of consumers, their purchasing behavior is more susceptible to the influence of power distance [30]. Therefore, power distance is more closely related to luxury purchases in studies on consumer behavior [38,39]. However, a wearable health care device is a health-related and life-oriented product that is not conspicuous. Therefore, power distance has a weak correlation with adoption intention toward wearable health care devices. This paper will not discuss the moderating effect of power distance on the relationships between perceived usefulness and perceived ease of use in adoption intention.

Second, people in a short-term orientation culture value technologies that bring usefulness to current life and work, while people in a long-term orientation culture value technologies that bring usefulness to future life [40]. Wearable health care devices are used not only by patients with chronic diseases [41,42] but also by healthy users for disease prevention [43]. Thus, the importance placed by people in both cultures on perceived usefulness depends on whether the concept is future-oriented or present-oriented. However, the measurement of this concept in the existing literature does not distinguish between these orientations [8,44]; thus, it is difficult to judge the moderating effect of long-term versus short-term orientations on the relationship between perceived usefulness and adoption intention. Moreover, since perceived ease of use is closely related to perceived usefulness [45], the moderating effect of long-term versus short-term orientation on the relationship between perceived ease of use and adoption intention also becomes difficult to judge. Therefore, this study does not analyze and test the moderating effects of long-term and short-term orientation.

#### Relationships Between Perceived Usefulness and Perceived Ease of Use in Adoption Intention

TAM illustrates the relationships between perceived usefulness and perceived ease of use in adoption intention [46]. Perceived usefulness refers to the degree to which people feel that using technology is helpful to their work and life [15]. Perceived ease of use refers to how much effort people need to use technologies [15]. The relationships between these variables and adoption intention have been proven in many studies related to technology adoption. For example, Hung et al [47] and Wu [48] showed that perceived usefulness and perceived ease of use positively affect the intention to adopt mobile commerce. In our research context, perceived usefulness is not only generally embodied in the improvement of work and life efficiency [13], it is specifically embodied in the improvement of the users’ health level [9,44]. These relationships regarding wearable health care devices have been confirmed in multiple studies [21,22]. Thus, we hypothesized the following:

Hypothesis 1a (H1a) and hypothesis 1b (H1b): perceived usefulness and perceived ease of use positively affect adoption intention toward wearable health care devices.

#### Moderating Effects of Individualism Versus Collectivism

Individualism versus collectivism reflects the degree to which people prefer to care for themselves and their families [30,37,49]. People in an individualistic culture put more emphasis on themselves, while people in a collectivist culture put more emphasis on their families [30,50]. Therefore, people in an individualistic culture value freedom and self-responsibility more and thus value their own health more [30]. This concern for health leads people in individualistic cultures to pay more attention to perceived usefulness of devices before purchase.

People in an individualistic culture are more accustomed to using emerging technologies such as email, online banking, and e-shopping in their daily lives. People from collectivist countries emphasize time spent with family and friends over time spent on the internet [30]. Therefore, people in an individualistic culture might have a higher frequency of using wearable health care devices. If the products are not easy to use, their experiences will be deeply affected. In addition, perceived ease of use positively affects the perceived usefulness of wearable health care devices [45] since perceived ease of use could help realize the function of the devices [51,52]. Moreover, people in an individualistic culture emphasize perceived usefulness more than people in a collectivist culture. Thus, people in an individualistic culture value perceived ease of use more, and we hypothesized the following:

Hypothesis 2a (H2a) and hypothesis 2b (H2b): The higher the degree of individualism, the higher the value placed on perceived usefulness (H2a) and perceived ease of use (H2b) toward adoption intention of wearable health care devices.

#### Moderating Effects of Masculinity Versus Femininity

Masculinity represents a preference for achievement, heroism, decisiveness, and material rewards for success, while femininity represents cooperation, humility, and quality of life [30]. The perceived usefulness of TAM emphasizes performance improvement and achievement, which is consistent with masculinity [53]. The meaning of achievement changes with time and context. In traditional societies, men pay attention to hunting and fighting, and in modern societies, men value economic achievement [30]. Regarding adoption intention for wearable health care devices, many people use them to measure sports achievements and enjoy competing with their peers [54]. Therefore, individuals in masculine cultures use wearable health care devices to satisfy their achievement motivation, and they value the perceived usefulness of the wearable health care device more.

People in masculine cultures hope to have challenging jobs to prove their competence and feel a sense of accomplishment, while people in feminine cultures hope to have a safer and higher quality life [30,37]. However, liking challenges does not mean that people in masculine cultures do not value perceived ease of use of wearable health devices. The greatest sense of accomplishment users get from wearable health care devices comes from recording their own sports achievements and competing with others [54] rather than showing they are good at using devices that are not easy to use. The increase in perceived ease of use contributes to the realization of functions of the device, such as functions of measurement, recording, and querying [45,51,52], which can effectively enhance the user’s sense of accomplishment. Because people in a masculine culture pay more attention to a sense of accomplishment than people in a feminine culture [30,37], people in a masculine culture also value perceived ease of use more, and we hypothesized the following:

Hypothesis 3a (H3a) and hypothesis 3b (H3b): The higher the degree of masculinity, the higher the value placed on perceived usefulness (H3a) and perceived ease of use (H3b) toward adoption intention of wearable health care devices.

#### Moderating Effects of Uncertainty Avoidance

People in a culture of high uncertainty avoidance value risk aversion more than people in a culture of low uncertainty avoidance [30]. The adoption of new technologies will bring about new risks, such as privacy risks [1] and imperfect technology [55,56]. This might make people in a high uncertainty avoidance culture hesitate to adopt new technologies. However, wearable health care devices can collect physical health data to control health risks, thereby making health conditions clearer and predictable [57], which is very attractive to people in a culture of high uncertainty avoidance. However, this does not mean that people in a high uncertainty avoidance culture will decide whether to adopt a wearable health care device based on its perceived usefulness. To reduce uncertainty, they are often prepared to engage in risky behavior [49] and are more impulsive [30]. For example, the higher the degree of uncertainty avoidance, the higher the maximum speed limit of a country (region) [30]. In addition, people in a high uncertainty avoidance culture have more concerns about health than people in a culture of low uncertainty avoidance [30]. Therefore, when faced with health-related decisions, people in a culture of high uncertainty avoidance are more likely to ignore meticulous thinking about the perceived usefulness of wearable health care devices and purchase products on impulse.

Regardless of whether people in a culture of high uncertainty avoidance consider the perceived usefulness when purchasing wearable health care devices, their purchase stems from health-related safety requirements [58]. Their need for safety takes precedence over other needs [30], such as the need for comfort and convenience represented by perceived ease of use. Therefore, people in a culture of high uncertainty avoidance pay less attention to the perceived ease of use of wearable health care devices than people in a culture of less uncertainty avoidance. Moreover, because perceived ease of use can improve the perceived usefulness of wearable health care devices [45,51,52] and people in a culture of low uncertainty avoidance are more concerned with perceived usefulness, people in a culture of low uncertainty avoidance perceived ease of use more, and we hypothesized the following:

Hypothesis 4a (H4a) and hypothesis 4b (H4b): The higher the degree of uncertainty avoidance, the less the value placed on perceived usefulness (H4a) and perceived ease of use (H4b) toward adoption intention of wearable health care devices.

#### Moderating Effects of Indulgence Versus Restraint

People in a culture of indulgence believe that enjoying life and entertainment are basic human needs, and natural desires should be satisfied [30]. People in a culture of restraint believe that human behavior should be restricted by social norms and prohibitions, and enjoying leisure activities, overconsumption, and similar indulgence behaviors are wrong [59]. Therefore, people in a high-indulgence culture are more likely to buy wearable health care devices because of the nonpractical functions of the products such as gamification [60] and innovation [61] rather than practical functions. A larger proportion of people in cultures with greater indulgence claim that their personal health is very good [49]. When people are more confident with their health conditions, they are less likely than people in cultures of restraint to consider perceived usefulness when deciding to purchase health products. Therefore, the greater the indulgence, the lower the value placed on perceived usefulness toward adoption intention of wearable health care devices.

Although people in a restraint culture value perceived usefulness more, and perceived ease of use determines the functional realization of wearable health care devices [45], people in an indulgence culture place more emphasis on perceived ease of use. This may be because people in an indulgence culture prefer pursuing the enjoyment of life [30] over spending time learning to use wearable health care devices. If a device is not easy to use, people in indulgence cultures are less likely to make the purchases. Conversely, people in a restraint culture are taught to be frugal and to limit their desires [30,37], and they believe the pursuit of pleasure is wrong [59]. Therefore, if the perceived usefulness of a device meets their requirements, they will buy and use a device regardless of perceived ease of use, and we hypothesized the following:

Hypothesis 5a (H5a) and hypothesis 5b (H5b): The greater the indulgence, the lower the value placed on perceived usefulness (H5a) and the higher the value placed on perceived ease of use (H5b) toward adoption intention of wearable health care devices.

## Methods

### Method Selection

Meta-analysis is a quantitative technique that generates a summary effect size for each relationship path [62]. This method has two functions. First, it helps scholars obtain a summary view of the results [63]. Second, this method is useful for hypothesis testing and moderator analysis [64]. This study used meta-analysis to explore the summary view of the relationships between perceived usefulness and perceived ease of use in adoption intention of wearable health care devices and the impact of the moderating effect of national culture on adoption intention. Therefore, the meta-analysis method is appropriate for this study.

### Data Sources and Search Strategy

We conducted a literature search by using keywords such as “wearable*,” “health*,” “fitness,” “wellness,” “medical,” “accept*,” “adopt*,” and “intention” to search for studies in the Web of Science, EBSCO, Engineering Village, China National Knowledge Infrastructure, IEEE Xplore, and Wiley Online Library databases. We then manually searched the references of the papers found for additional relevant titles to reduce the influence of publication bias.

### Selection Criteria

The study selection criteria were formulated considering the recommendations of Cooper [62] and the aim of this research. Studies included were empirical; reported sample size, correlation coefficient, and country of origin of the surveyed population; were related to adoption intention for wearable health care devices; and surveyed ordinary users and not nursing staff. Studies that did not use TAM or UTAUT as the main model, studies using continuance intention as the dependent variable (because the purpose of this paper is to promote the commercialization of devices rather than the maintenance of users after adoption), multiple studies using the same data (one of the studies would be retained in the paper), and review literature were excluded.

This article treats performance expectation, which belongs to UTAUT, as equivalent to the concept of perceived usefulness, which belongs to TAM. This article treats effort expectation, which belongs to UTAUT, as equivalent to the concept of perceived ease of use, which belongs to TAM. On one hand, other studies have regarded perceived usefulness and performance expectation [65-69] and perceived ease of use and effort expectation [69] as the same concept. On the other, the same results of multiple operations indicate that these operations focus on the same components and can enhance our confidence in the conclusions [62].

### Data Extraction

The extracted information included the first author’s name, year of publication, sample size, correlation coefficient matrix, and the location of the questionnaire collection. If the author did not report the location, we used the country (region) the authors came from. We got Hofstede’s cultural values by searching for that country (region) on the website of Hofstede’s cultural dimensions [70]. The required data were extracted independently by two researchers.

### Analysis Procedure

The meta-analysis consisted of 4 parts conducted using CMA (version 2.0, Biostat Inc) software. Funnel plots, Egger regression, and Rosenthal fail-safe N tests were used to determine whether publication bias existed [71,72]. The heterogeneity of various items was assessed using a Cochran Q test. When *P*<.05, the heterogeneity test was passed. We also calculated the *I*^2^ statistic, an indicator of heterogeneity in percentages [73].

Fixed-effects and random-effects models are the two main methods for calculating effect size [74]. We used the results of the heterogeneity test to select the appropriate model [73]. Because factors such as gender and age might affect the relationships between perceived usefulness and perceived ease of use in adoption intention [46], we used a random-effects model to calculate the main effect size. Sensitivity analysis was conducted to determine whether the elimination of any data item would influence the overall results. We conducted meta-regression analyses to estimate the moderating effects of national culture. For each regression, the correlation coefficient was the dependent variable and the value of the national culture dimension was the independent variable.

## Results

### Study Selection

**Figure 2:**
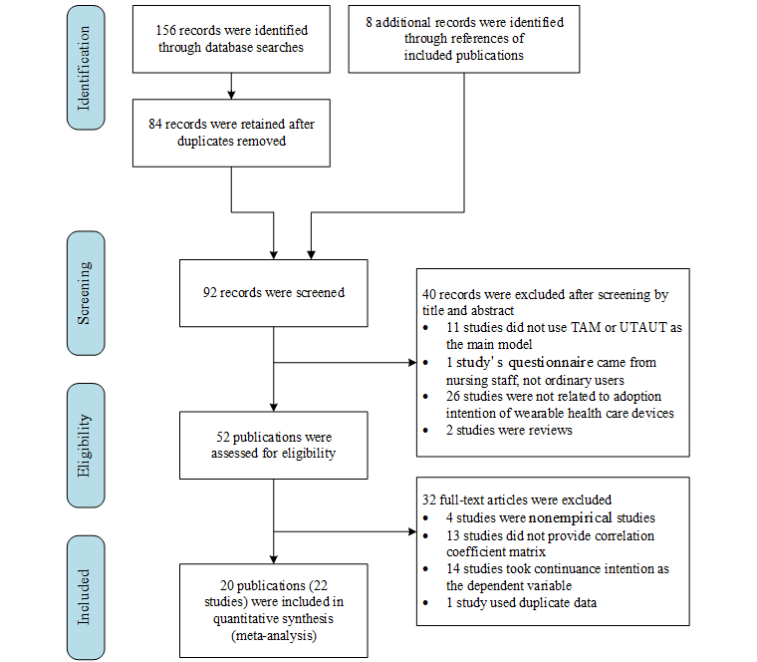
Preferred Reporting Items for Systematic Reviews and Meta-Analyses flowchart.

A total of 156 papers were found in our search on September 4, 2021. After deduplication, 84 remained, with 8 additional papers identified in the references. Next, 40 papers were excluded based on the titles and abstracts. After reading the full texts of the remaining 52 papers, we deleted 32 that did not meet the selection criteria, with a final total of 20 publications reporting on 22 effect sizes. Two of the 20 papers contained 2 studies. Therefore, 22 studies were included. Figure 2 shows the study flowchart with details.

### Study Characteristics Description

This meta-analysis included 20 publications [5-10,12,13,21,25,26,45,75-82] with 6128 participants. The 20 publications were conducted in 7 countries (regions) and published between 2015 and 2021. The sample size ranged from 100 [5] to 877 [13]. A total of 22 studies analyzed the relationship between perceived usefulness and adoption intention [5-10,12,13,21,25,26,45,75-82], and 18 studies analyzed the relationship between perceived ease of use and adoption intention [5-8,10,12,13,21,25,26,45,75,77,78,80,81,82], and 2 of the studies were from the same publication [25]. The characteristics of the included studies are presented in Multimedia Appendix 1.

### Meta-analysis

#### Publication Bias Test

**Figure 3:**
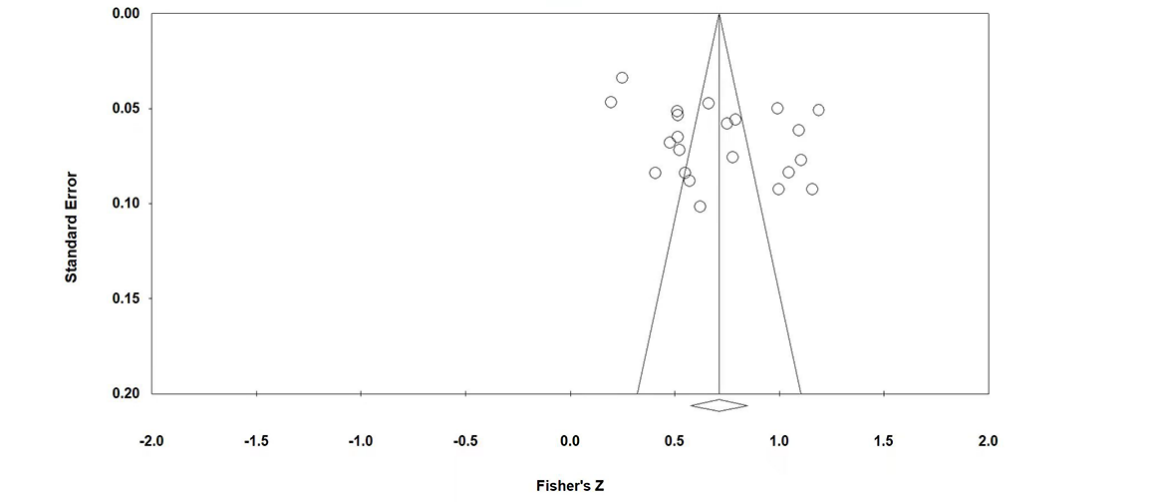
Funnel plot of studies on the perceived usefulness–adoption intention relationship.

**Figure 4:**
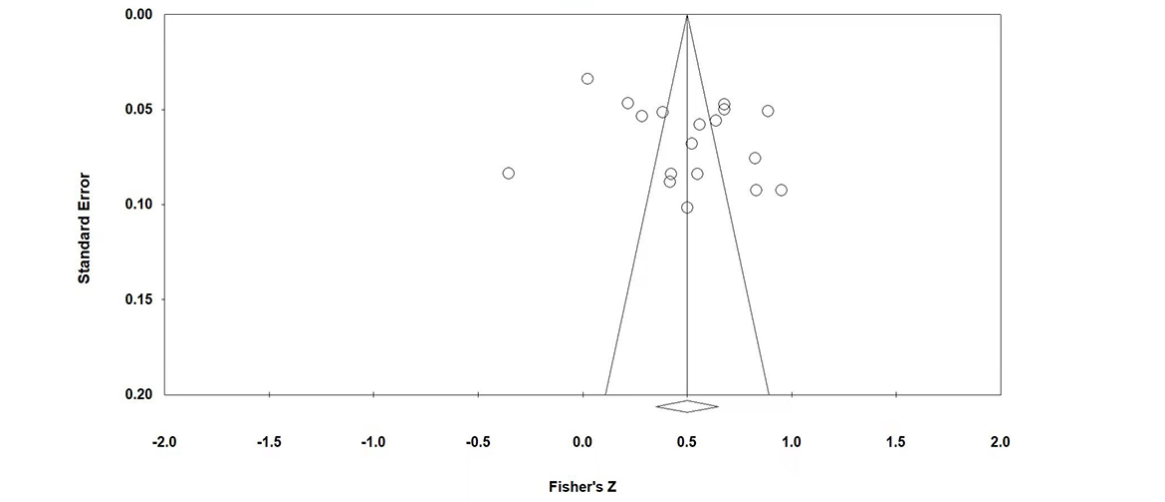
Funnel plot of studies on the perceived ease of use–adoption intention relationship.

The results of publication bias test are shown in Table 1, Figure 3, and Figure 4. According to the funnel plot, the studies on the perceived usefulness–adoption intention and perceived ease of use–adoption intention relationships were distributed on either side of the center lines, which indicates that the studies about these relationships do not have publication bias. If the Rosenthal fail-safe N is greater than 5M+10 (M is the number of research papers), publication bias does not exist. Table 1 shows that neither relationship had publication bias. According to the results of the Egger regression intercept, neither relationship had publication bias. Since no publication bias was found using 3 different tests, the main effect sizes of the meta-analysis are considered valid.

**Table 1:** Results of publication bias test.

Relationship	Rosenthal N	Egger regression intercept				
		Intercept	SE	LL^a^	UL^b^	*P* value
PU^c^-AI^d^	4967	7.489	3.784	–0.405	15.384	.06
PEOU^e^-AI	5047	5.973	4.116	–2.754	14.699	.17

^a^LL: lower limit.

^b^UP: upper limit.

^c^PU: perceived usefulness.

^d^AI: adoption intention.

^e^PEOU: perceived ease of use.

#### Heterogeneity Tests

Table 2 shows that the effect sizes of these studies are heterogeneous. Therefore, it is necessary to test the moderating effect. In addition, the random-effects model should be used when estimating the main effect size.

**Table 2:** Heterogeneity test results.

Relationship	Heterogeneity			
	Q	*df* (Q)	*P* value	*I* ^2^
PU^a^-AI^b^	598.249	21	<.001	96.490
PEOU^c^-AI	495.531	17	<.001	96.569

^a^PU: perceived usefulness.

^b^AI: adoption intention.

^c^PEOU: perceived ease of use.

#### Estimation of Main Effect Size

**Figure 5:**
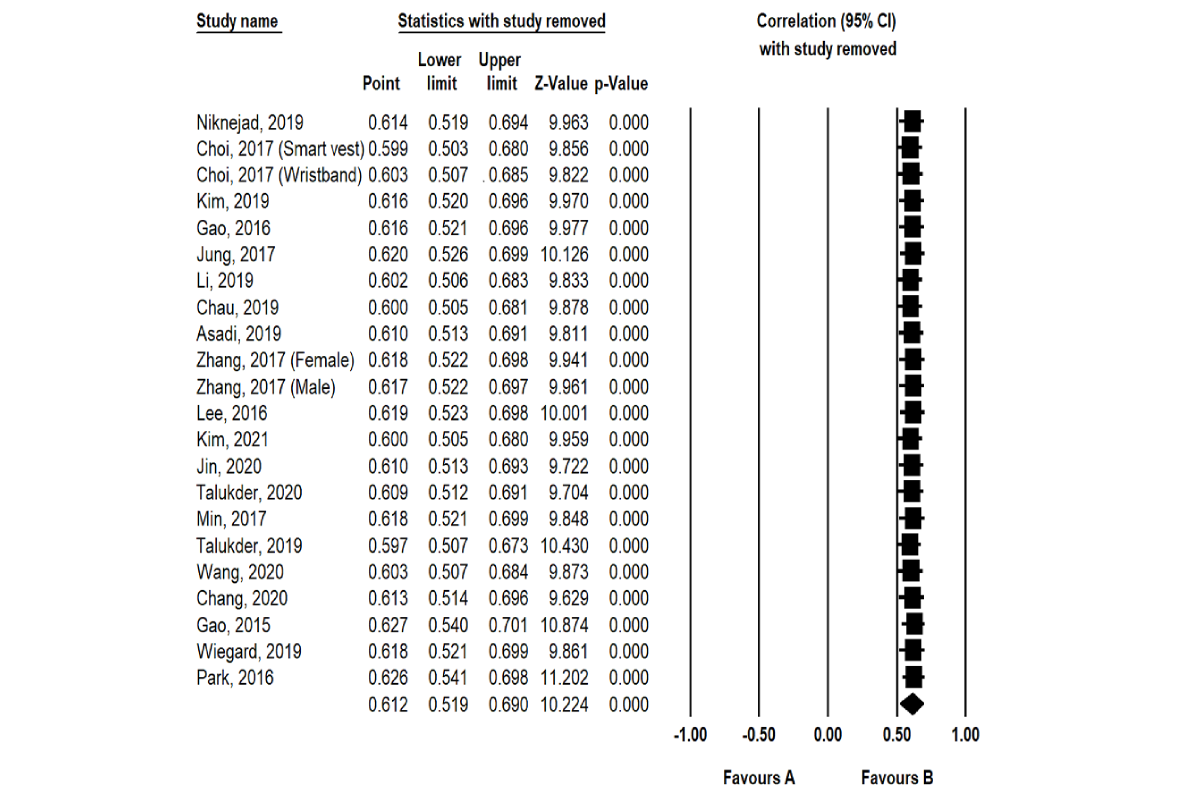
Sensitivity analysis results regarding the effect size of the perceived usefulness–adoption intention relationship.

**Figure 6:**
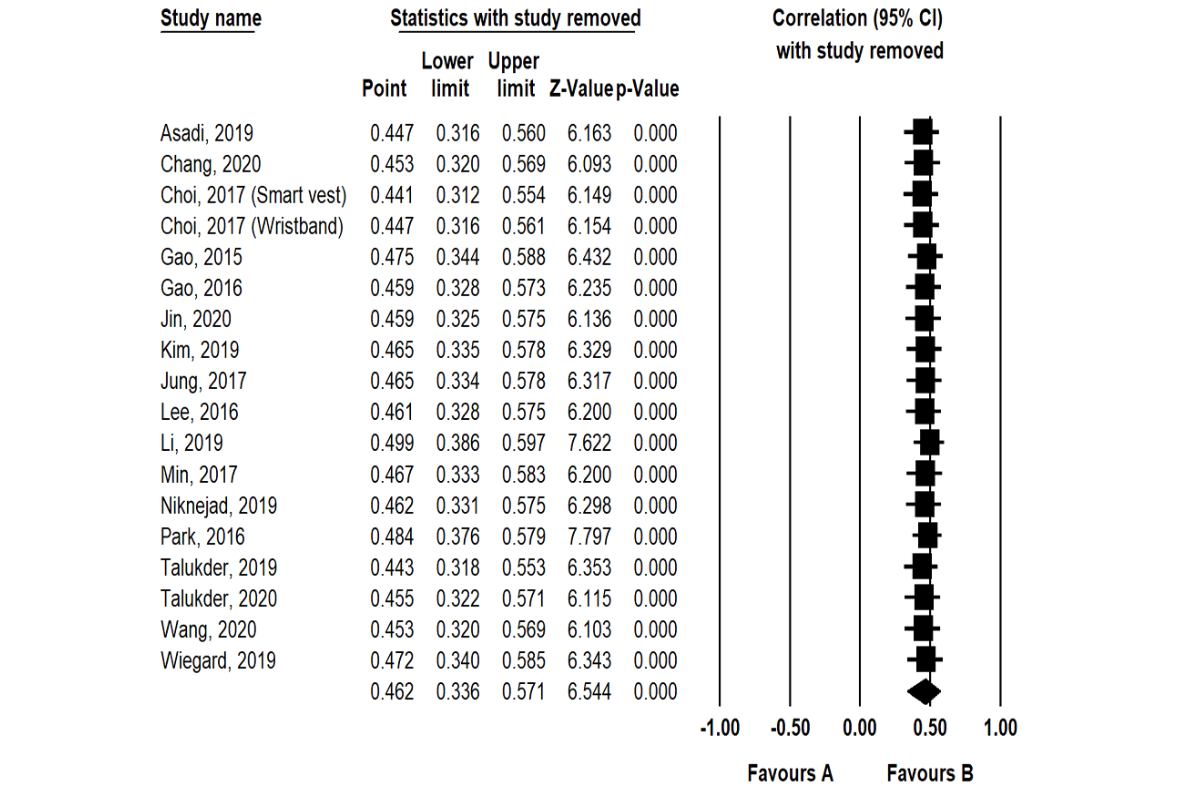
Sensitivity analysis results regarding the effect size of the perceived ease of use–adoption intention relationship.

The random-effects model was used to test the perceived usefulness–adoption intention and perceived ease of use–adoption intention relationships. Table 3 shows that the perceived usefulness–adoption intention (*r*=0.612, *P*<.001) and perceived ease of use–adoption intention (*r*=0.462, *P*<.001) relationships were significant. The correlation coefficients are both around 0.5, which means that the perceived usefulness–adoption intention and perceived ease of use–adoption intention relationships have moderately positive correlations [83]. In addition, the results of sensitivity analysis, presented in Figures 5 and 6, showed that the 2 correlation coefficients after any study removed fluctuates between 0.597 and 0.627 (perceived usefulness–adoption intention) and between 0.441 and 0.499 (perceived ease of use–adoption intention), indicating that the results of the meta-analysis have high stability. Therefore, these results confirm hypotheses H1a and H1b.

**Table 3:** Main effect size estimates.

Hypothesis	Relationship	*k*	Main effect size estimates									Supported
			Point estimate	95% CI			*Z*-value		*P* value			
				LL^a^	UL^b^							
H1a	PU^c^-AI^d^	22	0.612	0.519	0.690	10.224		<.001		Yes		
H1b	PEOU^e^-AI	18	0.462	0.336	0.571	6.544		<.001		Yes		

^a^LL: lower limit.

^b^UL: upper limit.

^c^PU: perceived usefulness.

^d^AI: adoption intention.

^e^PEOU: perceived ease of use.

#### Estimation of Moderating Effects of National Culture

The results are shown in Table 4. Individualism positively moderates the perceived usefulness–adoption intention (β=.003, *P*<.001) and the perceived ease of use–adoption intention (β=.003, *P*<.001) relationships. These results confirm hypothesis H2a and H2b. Masculinity positively moderates the perceived usefulness–adoption intention (β=.008, *P*<.001) and perceived ease of use–adoption intention (β=.006, *P*<.001) relationships. These results confirm hypotheses H3a and H3b. Uncertainty avoidance negatively moderates the perceived usefulness–adoption intention (β=–.005, *P*<.001) and perceived ease of use–adoption intention (β=–.004, *P*<.001) relationships. These results confirm hypotheses H4a and H4b. Indulgence positively moderates the perceived usefulness–adoption intention (β=.005, *P*<.001) and perceived ease of use–adoption intention (β=.009, *P*<.001) relationships. These results confirm hypothesis H5b but not hypothesis H5a.

**Figure 7:**
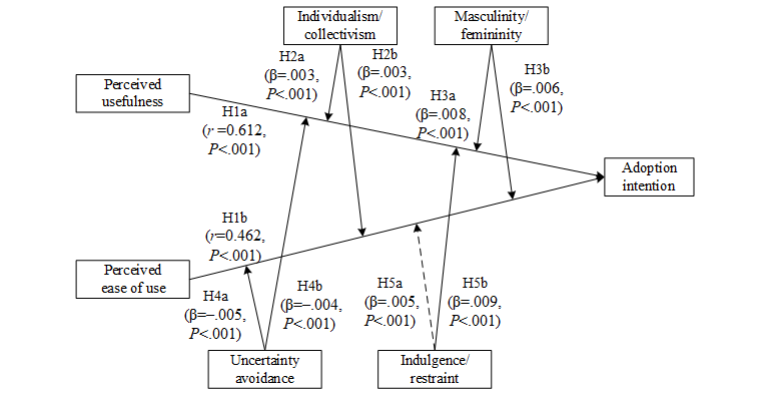
Meta-analysis results. H: hypothesis.

The results are summarized in Figure 7. The confirmed hypotheses are represented by a solid line, and the unproven hypotheses are represented by a dashed line.

**Table 4:** Results of moderating effects of national culture.

	Hypothesis	Relationship	Point estimate	SE	Lower limit	Upper limit	*Z*-value	*P* value	Supported
Individualism/collectivism									
	H3a	PU^a^-AI^b^	0.003	0.001	0.002	0.005	4.331	<.001	Yes
	H2b	PEOU^c^-AI	0.003	0.001	0.002	0.005	4.095	<.001	Yes
Masculinity/femininity									
	H3a	PU-AI	0.008	0.001	0.006	0.01	7.171	<.001	Yes
	H3c	PEOU-AI	0.006	0.001	0.004	0.008	5.588	<.001	Yes
Uncertainty avoidance									
	H4a	PU-AI	–0.005	0.001	–0.006	–0.004	–9.075	<.001	Yes
	H4b	PEOU-AI	–0.004	0.001	–0.005	–0.003	–7.721	<.001	Yes
Indulgence/restraint									
	H5a	PU-AI	0.005	0.001	0.003	0.007	5.124	<.001	No
	H5b	PEOU-AI	0.009	0.001	0.007	0.011	7.960	<.001	Yes

^a^PU: perceived usefulness.

^b^AI: adoption intention.

^c^PEOU: perceived ease of use.

## Discussion

### Findings on Main Effects

The results of this study showed that perceived usefulness (H1a) and perceived ease of use (H1b) positively affect adoption intention. These results are consistent with most of the literature on adoption intention in wearable health care devices [9,21]. The results are also consistent with the meta-analysis results in many other research contexts, such as mobile health service adoption [14] and mobile payment adoption [84]. Therefore, the relationships between perceived usefulness and perceived ease of use with adoption intention have once again proved to be robust. Moreover, the results for H1b can help clarify the debate on the relevance direction. This result does not support the uncorrelated result of the relationship between perceived ease of use and adoption intention [63]; thus, the relationship between these two variables should not be ignored in actual work.

### Findings on Moderating Effects of National Culture

Gender, age, voluntariness of use, and experience are important moderating variables in UTAUT [46], and gender and age are important in TAM3 [85]. The results of the moderating effects in this paper show that national culture also needs to be a focus in the research context of technology adoption, especially in the context of adoption intention in wearable health care devices. The specific conclusions are as follows:

The results on the moderating effect of individualism/collectivism found that individualism positively moderated the relationship between perceived usefulness and adoption intention (H2a) and the relationship between perceived ease of use and adoption intention (H2b). The test results of H2a and H2b are consistent with the results of Hung and Chou [31] and Zhang et al [86]. H2a states that people in individualistic cultures value personal health more [30], and thus the higher the degree of individualism, the higher the value placed on perceived usefulness toward adoption intention of wearable health care devices (H2a). However, this assumption ignores the fact that an important advantage of wearable health care devices is the implementation of health monitoring and reduction of health risks and costs [2]. People in a collectivist culture are willing to invest less income to maintain health compared to people in an individualistic culture [87]. From this point of view, people in a collectivist culture need devices to protect their health and reduce medical costs. The test result of H2a showed that the importance of mentioned facts in H2a is greater than that of ignored facts. Therefore, H2a is reasonable.

The results on the moderating effect of masculinity/femininity showed that masculinity/femininity positively moderates the influence of perceived usefulness (H3a) and perceived ease of use (H3b) on adoption intention. The test result of H3a is consistent with the findings of Hung and Chou [31], and both results are consistent with the findings of Zhang et al [86]. In our study, people in highly masculine cultures regard health achievements as an aspect of competition. This might be because health is a symbol of strength, which is consistent with the most essential masculine temperament [30]. The test result of H3b is contrary to the findings of Hung and Chou [31]. This result is possible as the perceived ease of use of technologies determines the user experience, and people in a feminine culture value the quality of life more [30]; therefore, people in this culture might value perceived ease of use more. However, when the impact of perceived usefulness on adoption intention is large enough, users who value perceived usefulness will also value perceived ease of use because the perceived ease of use of wearable health care devices could help realize the function of the devices [51,52]. Therefore, the test results of H3b are reasonable.

The results on the moderating effect of uncertainty avoidance showed that uncertainty avoidance negatively moderates the relationship between perceived usefulness (H4a) and perceived ease of use (H4b) with adoption intention. These results are consistent with those of Hung and Chou [31]. The test results for H4a are consistent with the findings of Yoon [88] and Lin [33]; neither study tested H4b. These results show that people in a culture of high uncertainty avoidance are indeed more likely to adopt technologies on impulse and then ignore the perceived usefulness and perceived ease of use of technologies. The negative moderating effect of uncertainty avoidance is easier to understand in this study since health is indeed an important thing for people in a high uncertainty avoidance culture [30] and might lead to irrational buying behaviors.

The results on the moderating effect of indulgence/restraint showed that indulgence strengthens the relationship between perceived ease of use and adoption intention (H5b); however, it does not weaken but strengthens the relationship between perceived usefulness and adoption intention (H5a). H5a states that people in indulgence cultures are less likely to value the perceived usefulness of wearable health care devices because people in such cultures are more likely to consider themselves healthy [30]. However, this reasoning process ignores the fact that people in an indulgence culture consume more junk food and are more obese [30]. In this regard, people in this culture need more wearable health care devices to monitor their health and encourage them to exercise. Thus, indulgence has a positive moderating effect. The test result of H5a showed that people in indulgence cultures rely more on the reality of their health condition when making decisions on adoption intention of wearable health care devices.

### Limitations

Our study has several limitations. First, this study focused only on the moderating effect of national culture on the relationship between the variables in TAM and adoption intention. However, the existing literature shows that trust [9-11], perceived privacy risk [1,12], customer innovation [9,13], and other variables affect people’s acceptance of wearable health care devices. Subsequent research should further explore the impact of national culture on the relationship between these variables and adoption intention. Second, this study does not discuss the moderating effect of national culture in different subgroups such as gender and age, classic moderating variables in TAM and UTAUT [46,85], because we were unable to obtain more detailed national cultural values of different genders and ages from the official website of Hofstede’s cultural dimensions [70]. However, these studies were necessary. For example, individualism is related to the income levels of individuals [30]. Therefore, the individualism scores of people of different ages in different countries might change when the world’s economic structure changes. Thus, it is necessary to conduct subgroup analysis of different ages.

### Implications for Practice

The results of this study could have implications for global wearable health care device providers and national health agencies. These results could help wearable health care device providers increase the adoption of the devices worldwide in two ways: guiding providers to develop more attractive and innovative devices by considering cultural factors and steering people toward wearable health care devices at the product sales stage. National health agencies can use these results to persuade people to use the devices for health management, conduct preventive treatment, and decrease medical expenses in the long term.

The application of these conclusions needs to target different national cultures. For example, for people in high masculinity cultures, such as Slovakia, Japan, and Hungary, health care device providers and national health agencies should pay more attention to perceived usefulness in the process of promoting the commercialization of wearable health devices.

When applying these conclusions, we should pay attention to not only the conclusions about the moderating effect of national culture but also the reasons for these conclusions. This can improve the efficiency of the persuasion process. For example, health care device providers and national health agencies should promote user adoption intention by emphasizing the perceived usefulness of the devices for potential users in a high masculinity culture and remind these potential users that they can compare their sport achievements with their peers for motivation using the devices.

### Conclusions

This meta-analysis provided comprehensive evidence for the positive relationships between perceived usefulness and perceived ease of use with adoption intention and the moderating effect of national culture on these relationships. Regarding the moderating effect, perceived usefulness and perceived ease of use have a greater impact on adoption intention for people in individualistic, masculine, low uncertainty avoidance and indulgence cultures, respectively.

## Appendix

Multimedia Appendix 1 Characteristics of the included studies.

## Abbreviations

TAM: technology acceptance model
UTAUT: unified theory of acceptance and use of technology
