# Supplementary material for: Impact of the Moderating Effect of National Culture on Adoption Intention in Wearable Health Care Devices: Meta-analysis
Source: JMIR Mhealth Uhealth. 2022 Jun 3;10(6):e30960. doi: 10.2196/30960 (PMC9206205; doi:10.2196/30960)
Supplement: Multimedia Appendix 1 [file mhealth_v10i6e30960_app1.docx]

## Multimedia Appendix 1

Characteristics of the included studies.

| **Number** | **Author, year** | **Variables** | **Correlation** | **Sample**  **size** | **Country**  **(region)** | **Individualism/**  **Collectivism** | **Masculinity/**  **Femininity** | | **Uncertainty Avoidance** | **Indulgence/**  **Restraint** |
| --- | --- | --- | --- | --- | --- | --- | --- | --- | --- | --- |
| 1 | Asadi, 2019[21] | PU | 0.651 | 178 | Malaysia | 26 | | 50 | 36 | 57 |
|  |  | PEOU | 0.678 |  |  |  |  |  |  |  |
| 2 | Chang, 2020[75] | PE | 0.580 | 452 | Taiwan, China | 17 | | 45 | 69 | 49 |
|  |  | EE | 0.590 |  |  |  |  |  |  |  |
| 3 | Chau, 2019[76] | PU | 0.802 | 171 | Hong Kong, China | 25 | | 57 | 29 | 17 |
| 4 | Choi, 2017[25]  (Smart vest) | PU | 0.760 | 120 | United States | 91 | | 62 | 46 | 68 |
|  |  | PEOU | 0.740 |  |  |  |  |  |  |  |
|  | Choi, 2017[25]  (Wristband) | PU | 0.820 | 120 | United States | 91 | | 62 | 46 | 68 |
|  |  | PEOU | 0.680 |  |  |  |  |  |  |  |
| 5 | Gao, 2015[7] | PE | 0.194 | 462 | China | 20 | | 66 | 30 | 24 |
|  |  | EE | 0.213 |  |  |  |  |  |  |  |
| 6 | Gao, 2016[10] | PU | 0.500 | 145 | China | 20 | | 66 | 30 | 24 |
|  |  | PEOU | 0.500 |  |  |  |  |  |  |  |
| 7 | Jin, 2020[77] | PE | 0.635 | 301 | South Korea | 18 | | 39 | 85 | 29 |
|  |  | EE | 0.507 |  |  |  |  |  |  |  |
| 8 | Kim, 2019[78] | PU | 0.516 | 132 | South Korea | 18 | | 39 | 85 | 29 |
|  |  | PEOU | 0.396 |  |  |  |  |  |  |  |
| 9 | Kim, 2021[79] | PE | 0.798 | 268 | Taiwan, China | 17 | | 45 | 69 | 49 |
| 10 | Jung, 2017[80] | PU | 0.387 | 145 | South Korea | 18 | | 39 | 85 | 29 |
|  |  | PEOU | 0.399 |  |  |  |  |  |  |  |
| 11 | Lee, 2016[81] | PE | 0.443 | 219 | South Korea | 18 | | 39 | 85 | 29 |
|  |  | EE | 0.480 |  |  |  |  |  |  |  |
| 12 | Li, 2019[8] | PU | 0.780 | 146 | China | 20 | | 66 | 30 | 24 |
|  |  | PEOU | -0.340 |  |  |  |  |  |  |  |
| 13 | Min, 2017[45] | PU | 0.470 | 380 | China | 20 | | 66 | 30 | 24 |
|  |  | PEOU | 0.365 |  |  |  |  |  |  |  |
| 14 | Niknejad, 2019[5] | PE | 0.553 | 100 | Malaysia | 26 | | 50 | 36 | 57 |
|  |  | EE | 0.462 |  |  |  |  |  |  |  |
| 15 | Park, 2016[13] | PU | 0.244 | 877 | South Korea | 18 | | 39 | 85 | 29 |
|  |  | PEOU | 0.023 |  |  |  |  |  |  |  |
| 16 | Talukder, 2019[6] | PE | 0.830 | 392 | China | 20 | | 66 | 30 | 24 |
|  |  | EE | 0.710 |  |  |  |  |  |  |  |
| 17 | Talukder, 2020[26] | PE | 0.658 | 325 | China | 20 | | 66 | 30 | 24 |
|  |  | EE | 0.564 |  |  |  |  |  |  |  |
| 18 | Wang, 2020[82] | PE | 0.758 | 406 | China | 20 | | 66 | 30 | 24 |
|  |  | EE | 0.590 |  |  |  |  |  |  |  |
| 19 | Wiegard, 2019[12] | PU | 0.473 | 353 | Germany | 67 | | 66 | 65 | 40 |
|  |  | EE | 0.276 |  |  |  |  |  |  |  |
| 20 | Zhang, 2017[9]  (Male) | PU | 0.480 | 197 | China | 20 | | 66 | 30 | 24 |
|  | Zhang, 2017[9]  (Female) | PU | 0.473 | 239 | China | 20 | | 66 | 30 | 24 |
